# Efficacy and sustainability of 40 Hz high‐definition transcranial alternating current stimulation (tACS) in the treatment of sleep disturbances in mild neurocognitive disorders due to Alzheimer’s disease

**DOI:** 10.1002/alz.094951

**Published:** 2025-01-09

**Authors:** Hanna Lu, Jing Li, Xi Ni, Natalie Shu Yang, Yun Kwok Wing, Suk Ling Ma, Linda Chiu Wa Lam, Li Zhang

**Affiliations:** ^1^ Centre for Neuromodulation and Rehabilitation, The Affiliated Brain Hospital of Guangzhou Medical University, Guangzhou, Guangdong China; ^2^ The Chinese University of Hong Kong, Hong Kong Hong Kong; ^3^ The Chinese University of Hong Kong, Hong Kong, Hong Kong Hong Kong; ^4^ University of Greifswald, Greifswald Germany

## Abstract

**Background:**

To investigate the effects of 40 Hz high‐definition transcranial alternating current stimulation (HD‐tACS) on subjective sleep quality and domain‐specific cognitive functions in mild neurocognitive disorders due to Alzheimer’s disease (NCD‐AD).

**Method:**

This study was a double blind, sham‐controlled randomized clinical trial. Ninety‐nine mild NCD‐AD patients were randomly assigned to receive a 4‐week course treatment of either 40 Hz HD‐tACS, HD transcranial direct current stimulation (HD‐tDCS), or sham transcranial current stimulation (HD‐tCS). Global cognition was assessed by Montreal Cognitive Assessment (MoCA). Subjective sleep quality was assessed by Pittsburgh Sleep Quality Index (PSQI). PSQI total score>5 was defined as sleep disturbances.

**Result:**

The mean score of PSQI was 11.7 in mild NCD‐AD patients. Repeated sessions of 40 Hz HD‐tACS and HD‐tDCS treatments significantly enhanced the subjective sleep quality and cognition. Compared with HD‐tDCS, the individuals who received 40 Hz HD‐tACS had more improvement on sleep quality (score changes of PSQI: 6.01 vs. 3.72, p<0.001) and cognitive function (MoCA: 2.5 vs.1.5, p<0.01) during a 2‐month follow‐up period.

**Conclusion:**

Mild NCD‐AD patients with sleep disturbances who received 40 Hz HD‐tACS had pronounced enhancement in subjective sleep quality and global cognition. To identify novel modality of transcranial brain stimulation is important toward an effective strategy for the comorbidities in preclinical AD.

